# Clinical practice of analysis of anti-drug antibodies against interferon beta and natalizumab in multiple sclerosis patients in Europe: A descriptive study of test results

**DOI:** 10.1371/journal.pone.0170395

**Published:** 2017-02-07

**Authors:** Jenny Link, Ryan Ramanujam, Michael Auer, Malin Ryner, Signe Hässler, Delphine Bachelet, Cyprien Mbogning, Clemens Warnke, Dorothea Buck, Poul Erik Hyldgaard Jensen, Claudia Sievers, Kathleen Ingenhoven, Nicolas Fissolo, Raija Lindberg, Verena Grummel, Naoimh Donnellan, Manuel Comabella, Xavier Montalban, Bernd Kieseier, Per Soelberg Sørensen, Hans-Peter Hartung, Tobias Derfuss, Andy Lawton, Dan Sikkema, Marc Pallardy, Bernhard Hemmer, Florian Deisenhammer, Philippe Broët, Pierre Dönnes, Julie Davidson, Anna Fogdell-Hahn, ABIRISK Consortium

**Affiliations:** 1 Department of Clinical Neuroscience, Karolinska Institutet, Stockholm, Sweden; 2 KTH – Royal Institute of Technology, Stockholm, Sweden; 3 Innsbruck Medical University, Innsbruck, Austria; 4 CESP, Université Paris-Sud, UVSQ, INSERM, Université Paris-Saclay, Villejuif, France; 5 Medical Faculty, Department of Neurology University of Düsseldorf, Düsseldorf, Germany; 6 Department of Neurology, Technische Universität München, Munich, Germany; 7 Danish Multiple Sclerosis Center, Rigshospitalet, University of Copenhagen, Copenhagen, Denmark; 8 University Hospital Basel, Basel, Switzerland; 9 Centre d'Esclerosi Múltiple de Catalunya (Cemcat), Hospital Universitari Vall d'Hebron, Barcelona, Spain; 10 Ipsen Biopharm Ltd, Berkshire, United Kingdom; 11 GlaxoSmithKline R&D, Uxbridge, Middlesex, United Kingdom; 12 INSERM UMR 996, Univ. Paris-Sud, Faculty of Pharmacy, Université Paris-Saclay, Châtenay-Malabry, France; 13 Assistance Publique - Hôpitaux de Paris, Hôpital Paul Brousse, Villejuif, France; 14 SciCross AB, Skövde, Sweden; Leiden University Medical Center, NETHERLANDS

## Abstract

Antibodies against biopharmaceuticals (anti-drug antibodies, ADA) have been a well-integrated part of the clinical care of multiple sclerosis (MS) in several European countries. ADA data generated in Europe during the more than 10 years of ADA monitoring in MS patients treated with interferon beta (IFNβ) and natalizumab have been pooled and characterized through collaboration within a European consortium. The aim of this study was to report on the clinical practice of ADA testing in Europe, considering the number of ADA tests performed and type of ADA assays used, and to determine the frequency of ADA testing against the different drug preparations in different countries. A common database platform (tranSMART) for querying, analyzing and storing retrospective data of MS cohorts was set up to harmonize the data and compare results of ADA tests between different countries. Retrospective data from six countries (Sweden, Austria, Spain, Switzerland, Germany and Denmark) on 20,695 patients and on 42,555 samples were loaded into tranSMART including data points of age, gender, treatment, samples, and ADA results. The previously observed immunogenic difference among the four IFNβ preparations was confirmed in this large dataset. Decreased usage of the more immunogenic preparations IFNβ-1a subcutaneous (s.c.) and IFNβ-1b s.c. in favor of the least immunogenic preparation IFNβ-1a intramuscular (i.m.) was observed. The median time from treatment start to first ADA test correlated with time to first positive test. Shorter times were observed for IFNβ-1b-Extavia s.c. (0.99 and 0.94 years) and natalizumab (0.25 and 0.23 years), which were introduced on the market when ADA testing was already available, as compared to IFNβ-1a i.m. (1.41 and 2.27 years), IFNβ-1b-Betaferon s.c. (2.51 and 1.96 years) and IFNβ-1a s.c. (2.11 and 2.09 years) which were available years before routine testing began. A higher rate of anti-IFNβ ADA was observed in test samples taken from older patients. Testing for ADA varies between different European countries and is highly dependent on the policy within each country. For drugs where routine monitoring of ADA is not in place, there is a risk that some patients remain on treatment for several years despite ADA positivity. For drugs where a strategy of ADA testing is introduced with the release of the drug, there is a reduced risk of having ADA positive patients and thus of less efficient treatment. This indicates that potential savings in health cost might be achieved by routine analysis of ADA.

## Introduction

Immunogenicity of biotechnology-derived proteins (BPs) is of increasing concern in modern medicine, but there are different opinions on whether testing for anti-drug antibodies (ADA) should be an integrated part of clinical routine or not. The different standpoints have ranged from that ADA testing adds essential information used for treatment decision, to that the reappearance of clinical symptoms would lead to a change of treatment regardless of ADA testing. Since ADA can develop neutralizing characteristics that reduces the efficacy of a drug, it is important to have the tools ready to assess the potential immunogenicity of new drugs released on the market. ADA testing is also of importance for determining the immunogenicity and safety profiles in clinics in order to issue guidelines on how to give patients the best treatment scheme while also making sure that the money spent by the clinics is used efficiently.

For treatment of multiple sclerosis (MS), some European countries, but not all, have had ADA testing as an integrated part of clinical practice. The disease modifying drug interferon beta (IFNβ) is a first-line treatment in MS that reduces the relapse rate, new lesion formation and disability accumulation over time [1–3]. There are two different types of IFNβ products: IFNβ-1a that is given either intramuscular (i.m.) at 30 μg once weekly (Avonex^®^, released in Europe in 1997) or subcutaneous (s.c.) at 22 μg or 44 μg three times weekly (Rebif^®^, released in Europe in 1998), and the two identical IFNβ-1b preparations given s.c. at 250 μg every other day (Betaferon^®^ and Extavia^®^, released in Europe in 1995 and 2008 respectively). The immunogenicity is known to differ between these preparations and this could be influenced by various drug- and patient-related factors, e.g. dosing and frequency, aggregate content and genetic background [4,5]. Neutralizing ADA (NAb) develop in up to 47% of patients using IFNβ-1b and up to 28% and 6% for those treated with s.c. IFNβ-1a and i.m. IFNβ-1a, respectively [4].

MS patients have been treated with IFNβ since the 1990s, but the clinical practice of testing for ADA and NAbs against the drug varies between countries. In Denmark it was a mandatory part of the treatment regime from the beginning in 1996, while in other countries it is only used when there is an indication of treatment failure. In Sweden the tests were provided for free between 2003 and 2005 and later paid by the clinics. In Austria it was paid by the clinics from the beginning and in other countries (Switzerland, Spain and Germany) testing has only been provided as part of research projects. Different ADA assays have been used and these have varied over time, even within a country. Austria, Denmark, Spain, Switzerland and Sweden have used NAb tests both for screening and titration. In Germany all patients were first screened with an enzyme linked immunosorbent assay (ELISA)-based ADA test and then confirmed and titrated with a NAb test.

When the monoclonal antibody natalizumab (Tysabri^®^) became available as a treatment for MS after reintroduction in 2006, a test for ADA was developed by Biogen Idec (Maine Biotechnology Services Inc., Portland, ME, USA) and provided to most countries from the beginning. The same bridging ELISA method for ADA detection was used in all laboratories included in this study, and yearly controls of 20 blinded test samples were used to ensure equal performance of the test in different laboratories. The cut-point for ADA positivity in this assay is set to a level that corresponds to neutralizing capacity of the antibodies [6], and thus considered as a “semi-proxy” for a NAb assay. Using this assay, anti-natalizumab ADA has been reported to develop in 4.5–12% of treated patients [6,7]. Assays for measuring drug levels of IFNβ or natalizumab have not been used in any of the countries.

Data on ADA and NAb have been collected over several years in Europe, but a systematic comparison between countries regarding number of patients and tests, and use of different drug preparations over time has not been performed previously. Here we present the most extensive amount of post-marketing immunogenicity data for MS patients treated with IFNβ and natalizumab in Europe. Collaborative analysis of this data may allow new insights into clinical relevance of ADA testing, in how ADA monitoring has been integrated in treatment decisions, and what may be the unmet future needs in immunogenicity related research.

Hence, the objective of this study was to integrate all retrospective ADA data available to the Anti-Biopharmaceutical Immunization: prediction and analysis of clinical relevance to minimize the RISK (ABIRISK)-EU consortium in the MS field (www.abirisk.eu) in a common database, describing and comparing results from monitoring of ADA in MS patients treated with IFNβ or natalizumab, and to report on type of ADA assay used over time, time of sample collection for analysis, and treatment duration and age at time of testing.

## Materials and methods

### Collection of data

Retrospective data on ADA tested patients were collected from seven cohorts in Europe and standardized to a common data format loosely based on the CDISC standards (www.cdisc.org). Where no previous variable description could be found in CDISC, a local variable description was used. All variables were described in a data load plan that was used by all data custodians when preparing data. Anonymized data from the different cohorts was merged and uploaded into the translational data analysis platform tranSMART [8]. For advanced analysis, data was exported and analyzed using R [9]. All sites requiring had approval from local ethics committees for uploading anonymized data.

The uploaded data from 20,695 patients and 42,555 samples contained both patient specific demographics such as age, gender, date of first IFNβ prescription, whether the patient was treatment naive at first test for ADA, treatment information at each date of sampling, type of assay used, and test result (i.e. negative/positive/titer level). After quality control of the data a total of 20,115 patients and 41,339 samples remained for analysis (S1 Fig)

### Description of cohorts

#### Sweden

Testing for IFNβ NAb was initiated 2003 in Sweden, and clinics from all over the country were encouraged to sample all patients on IFNβ treatment. In 2010 it was recommended, based on the publication by Polman et al, that switching to a non-IFNβ treatment should be considered if a NAb positive test with a titer above 600 ten-fold reduction units per milliliter (TRU/ml) was registered [10]. Testing for ADA against natalizumab was initially free of charge (courtesy of Biogen Idec) in Sweden between 2006 and 2011, and clinics were encouraged to send samples for ADA testing at baseline before first infusion and every six months following treatment start. All samples were sent to one nationwide reference laboratory at Karolinska Institutet in Stockholm, and data on all ADA-tested samples in Sweden between 2001 and 2013 are included in this study. For the Swedish cohort the study was approved by the regional ethical committee in Stockholm, Sweden, and approval for export of data from the national Multiple Sclerosis Registry (www.neuroreg.se) was given by the Research Board of the Swedish MS Society.

#### Austria

Throughout Austria, testing for NAbs against IFNβ was initiated 1995 and ADA testing of natalizumab- treated patients was introduced in 2006. All samples were sent for ADA testing at the discretion of doctors to a reference laboratory in Innsbruck where testing for ADA was part of a scientific project supported by unrestricted grants from industry. Results from all samples tested between 1995 and 2014 are included in this study. Retrospective analyses based on anonymous data that have been collected during routine procedures do not require ethical approval in Austria.

#### Denmark

In Denmark, IFNβ NAb testing has been part of the clinical routine since 1996 and ADA test for natalizumab was also introduced in 2007. For this study, only results from IFNβ NAb tests performed with the same Luciferase assay method between 2009 and 2014 were included. Before 2009 IFNβ NAb was analysed by Klaus Bendtzen, Rigshospitalet, and later by Biomonitor. At Rigshospitalet, an ELISA was used to screen for ADA from 2005 to 2009, and for confirmation of ADA from 2009 to 2012. Analysis with the myxovirus resistance protein A (MxA) gene expression assay (MGA) for final confirmation of ADA-positive samples before cessation of IFNβ treatment was an option used from 2005. The MGA was calculated as an index based on the expression of MxA and the endogenous control GAPDH, with NAb-positive samples having a relative MxA expression less than 5 and NAb-negative above 15. Samples tested for ADA against natalizumab were analyzed after 2007. Only data from patients who approved participation in the Danish Multiple Sclerosis Biobank were included from 2007–2014. Patients and samples for which important information were missing was excluded. For IFNβ, the included data constituted 39% and 42% of the total number of patients and measured samples, respectively. For natalizumab, 73% of patients and 89% of total measured samples were included. All tests were performed in one central reference laboratory at Rigshospitalet in Copenhagen. The Danish MS cohort collected in the Danish Multiple Sclerosis Biobank was approved by the regional scientific ethical committee in Copenhagen and Frederiksberg, Denmark, and approval for use of data was given by the Danish Data Protection Agency in the Capital Region, Denmark.

#### Germany

In Germany, a two-tiered testing approach was used in which samples were first screened for ADA against IFNβ and positive samples were subsequently tested for NAbs against IFNβ. Results from ADA tests against natalizumab were not available for this study.

In Dusseldorf, test results and anonymized data from the German central laboratory for NAb testing were included in this study. To ensure similar testing protocols for all samples, only data from 2008 to 2014 were included. Analysis was performed at the discretion of the treating physician, and included follow-up testing.

In Munich, ADA and NAb testing of IFNβ started in 2003 and all patients tested until 2008 are included in this study. The data from Munich were originated from the German reference laboratory with a nationwide collection and all samples included in this cohort were part of a research project. One sample per patient was provided from this cohort. For the Munich cohort the study was approved by the ethical committee of the School of Medicine of the Technical University of Munich, Germany.

#### Switzerland

In Switzerland, samples from IFNβ-treated patients with a suspicion of NAbs were sent to a reference laboratory in Italy for NAb testing (Dr. Bertolotto; Centro di Riferimento Regionale Sclerosis Multipla, Orbassano, Turin, Italy). A collection of patients with samples tested between 2005 and 2009 was part of this study. No results from testing for ADA against natalizumab were provided. The use of anonymous data for retrospective analysis did not require ethical approval in Switzerland at the time of sampling.

#### Spain

Spanish MS patients included in the study were tested for NAb against IFNβ between 1996 and 2005. No data on anti-natalizumab ADA testing was provided. The Spanish cohort was collected in a research laboratory at the Centre d'Esclerosi Múltiple de Catalunya (Cemcat) in Barcelona, and two tested samples per patient were provided. For the Spanish cohort the study was approved by the Hospital Vall d’Hebron ethical committee in Barcelona, Spain.

### ADA testing

Capture ELISA for testing of ADA against IFNβ was used in the German cohorts and served as a screening method to identify which samples to test for NAbs [11]. Reactivity above 25% of the highest positive control was considered to be antibody positive in the ELISA.

ADA testing for natalizumab was performed in Austria, Denmark and Sweden using the same validated protocol and standardized bridging ELISA method developed by Biogen Idec as previously described [6]. Samples were considered positive if antibody levels equal to or above 0.5 μg/mL were detected and further that the ratio of absorbance optical density values between background and sample was less than 0.5. In this assay, positivity is set at a level corresponding to reduced efficacy of the drug and discontinued treatment is recommended if patients are confirmed to be persistently positive, i.e. two positive samples taken at least six weeks apart [6]. For this study, any time positive after treatment initiation was considered as a positive ADA result.

### NAb testing

**Fig 1:**
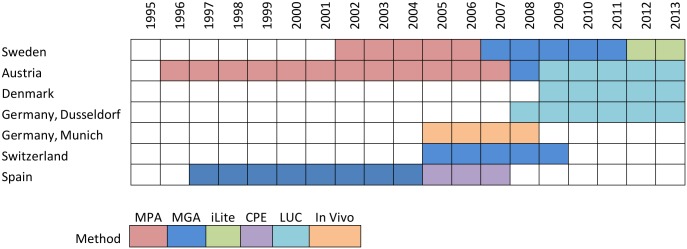
Main methods used for NAb testing in different cohorts over time. In cases where two different methods were used in the same year, only the most frequently used method is shown.

Samples tested for NAbs against IFNβ have been analyzed using different methods and protocols, both within cohorts over time and between cohorts (Fig 1).

NAb testing with the MxA protein expression assay (MPA) or MGA were used in Sweden, Austria and Switzerland [12–14]. MGA was also used in Spain for a few samples during 1997 and 2003 (not shown in Fig 1) but the majority of samples were analyzed by the CPE method [15]. Austria, Denmark and Dusseldorf used the Luciferase (LUC) assay according to previously described protocols [16–18]. In Munich the In Vivo assay was used, in which the biological in vivo activity of IFNβ was measured by induction of MxA gene expression and compared to newly treated antibody-negative control donors [19]. A cross-validation study on 247 samples was performed between Austria and Munich using the MGA assay that showed an agreement of 96% for classifying samples as NAb positive (>30 TRU/ml) versus NAb negative (personal communication with F. Deisenhammer). Only in Sweden was the iLite anti-human IFNβ-1a bioassay used for the latest samples [20]. Over 95% correlation between NAb titer levels derived from the former MGA and MPA methods as well as between MGA and iLite methods has been shown in Sweden. When classifying samples as NAb positive (>10 TRU/ml) versus NAb negative, the iLite assay showed lower sensitivity by only detecting 16 of the 23 positive samples in the MGA [21]. The Kawade formula was used to adjust NAb titers obtained with the MPA, MGA, LUC and iLite methods [22]. Titers or neutralizing capacity for positivity in each NAb assay is presented in Table 1. In all analyses, a positive sample was defined according to the cut-off for NAb positivity used in the respective cohorts, as shown in Table 1. Unless otherwise specified, all results regarding anti-IFNβ ADA were analyzed using NAb tests. Any time positive was considered as NAb positive in this study. IFNβ-1a s.c. given in doses 22 μg or 44 μg were analyzed together.

**Table 1:** Definition of positive samples according to the cut-off for NAb positivity in different cohorts. For MPA, MGA, iLite, CPE and LUC assay the cut-off values are presented in ten-fold reduction units per milliliter (TRU/ml). For the In Vivo assay, a sample is considered positive for biologically active antibodies if the MxA induction is decreased by more than 50% compared to antibody-negative controls.

Method/cohort	Negative	Positive	Medium positive	High positive
**Sweden**				
MPA	< 10 TRU/ml	≥ 10 TRU/ml	≥ 50 TRU/ml	≥ 200 TRU/ml
MGA	< 10 TRU/ml	≥ 10 TRU/ml	≥ 50 TRU/ml	≥ 200 TRU/ml
iLite	< 10 TRU/ml	≥ 10 TRU/ml	≥ 50 TRU/ml	≥ 200 TRU/ml
**Austria**				
MPA	< 20 TRU/ml	≥ 20 TRU/ml	-	≥ 100 TRU/ml
MGA	< 20 TRU/ml	≥ 20 TRU/ml	-	≥ 100 TRU/ml
LUC	< 20 TRU/ml	≥ 20 TRU/ml	-	≥ 100 TRU/ml
**Denmark**				
LUC	< 20 TRU/ml	≥ 20 TRU/ml	-	-
**Dusseldorf**				
LUC	< 20 TRU/ml	≥ 20 TRU/ml	-	> 100 TRU/ml
**Munich**				
In Vivo	< 50% decrease	≥ 50% decrease	-	-
**Spain**				
CPE	< 20 TRU/ml	≥ 20 TRU/ml	-	> 100 TRU/ml
**Switzerland**				
MGA	< 20 TRU/ml	≥ 20 TRU/ml	-	-

### Statistics

Data was processed using standard workflows wherein patients’ visits and tests were categorized according to information available regarding treatment, date, patient age, treatment start date, etc. Descriptive statistics included calculation of medians and counts of various data subsets. Fisher’s exact test was used to test association for statistical significance within groups at the 0.05 level when estimating the risk of becoming ADA positive on natalizumab if previously NAb positive against IFNβ. When testing for sample numbers across ages, the significance of the slope of the regression line was assessed.

Calculations were made using R 3.2 [23] as well as Python 2.7 and Pandas 0.13.1. All box plots and graphs were made with matplotlib 1.3.1.

## Results

### Overall number of ADA tests against IFNβ and natalizumab

**Fig 2:**
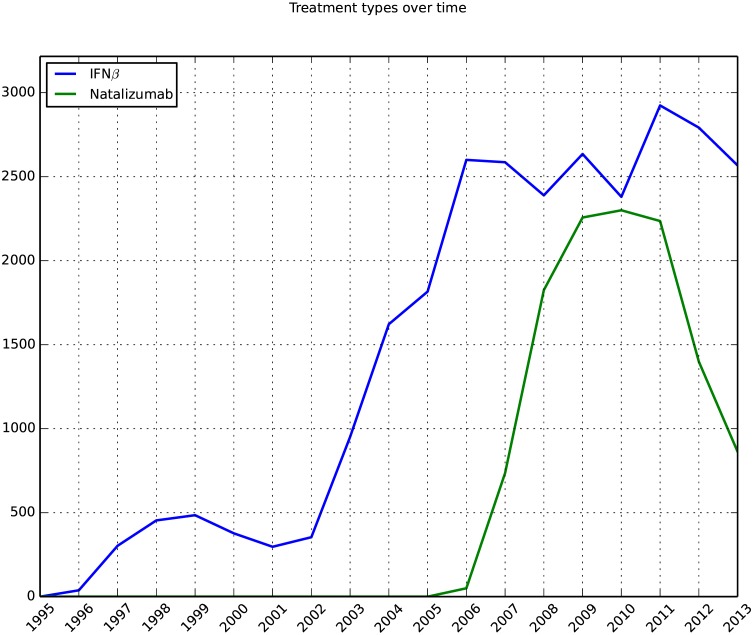
Overall number of ADA tests against IFNβ and natalizumab over time. This includes all tested samples from every cohort and not only samples from those cohorts that have data during the entire period of investigation.

The number of samples from MS patients that received IFNβ treatment and were analyzed for ADA increased from 2002 to 2006 and remained stable with around 2500 samples each year over the subsequent period of investigation. For natalizumab-treated patients that were tested for ADA the number of samples increased from 2006 to 2010, when over 2000 samples were analyzed, and then declined to less than 1000 samples between year 2011 and 2013 (Fig 2).

### Comparison of number of ADA tests per patient between countries

A mean of 2.05 samples were tested per patient in all countries and ranged from 1.0 in Munich to 2.8 in Denmark, reflecting the selection of data included in this study. Details for each country are shown in Table 2. Approximately half of the patients only had one test, giving a median of 1 test per patient. The median varied between countries: 2 in Sweden (max = 13), 1 in Austria (max = 50), 3 in Denmark (max = 9), 1 in Dusseldorf (max = 7), and 1 in Munich (max = 1).

**Table 2:** Description of cohorts.

Country/Cohort	Patients (N)	Male/ Female	% Female	Samples (N)	Max tests per patient (N)	Mean tests per patient	Median tests per patient
**Austria**	4582	1377/3158^1^	69.6%	12351	50	2.7	1.0
**Denmark**	1936	566/1370	70.8%	5517	9	2.8	3.0
**Germany/ Dusseldorf**	5498	1565/3912^2^	71.4%	6677	7	1.2	1.0
**Germany/ Munich**	2255	635/1616^3^	71.8%	2255	1	1.0	1.0
**Spain**	64	22/42	65.6%	128	2	2.0	2.0
**Sweden**	6189	1753/4436	71.7%	15383	13	2.5	2.0
**Switzerland**	201	61/140	69.7%	238	3	1.2	1.0

^1^ 47 did not have information on gender

^2^ 21 did not have information on gender

^3^ 4 did not have information on gender

An extreme case of 50 tests in Austria represents monitoring of a patient that switched from IFNβ to natalizumab. This patient had nine NAb-negative samples when treated with IFNβ-1a s.c. (7 tests) and IFNβ-1b-Betaferon (2 tests), with one borderline positive test. After switching to natalizumab, the initial test after treatment start was positive for anti-natalizumab ADA. Forty additional tests during treatment with natalizumab taken during the next five and a half years were all ADA negative.

When examining the number of tests performed and age at sampling, there was no significant deviation of the regression line from slope = 0. Therefore, sample numbers were consistent across all ages.

### Time from treatment start to first tested sample versus first positive sample

**Fig 3:**
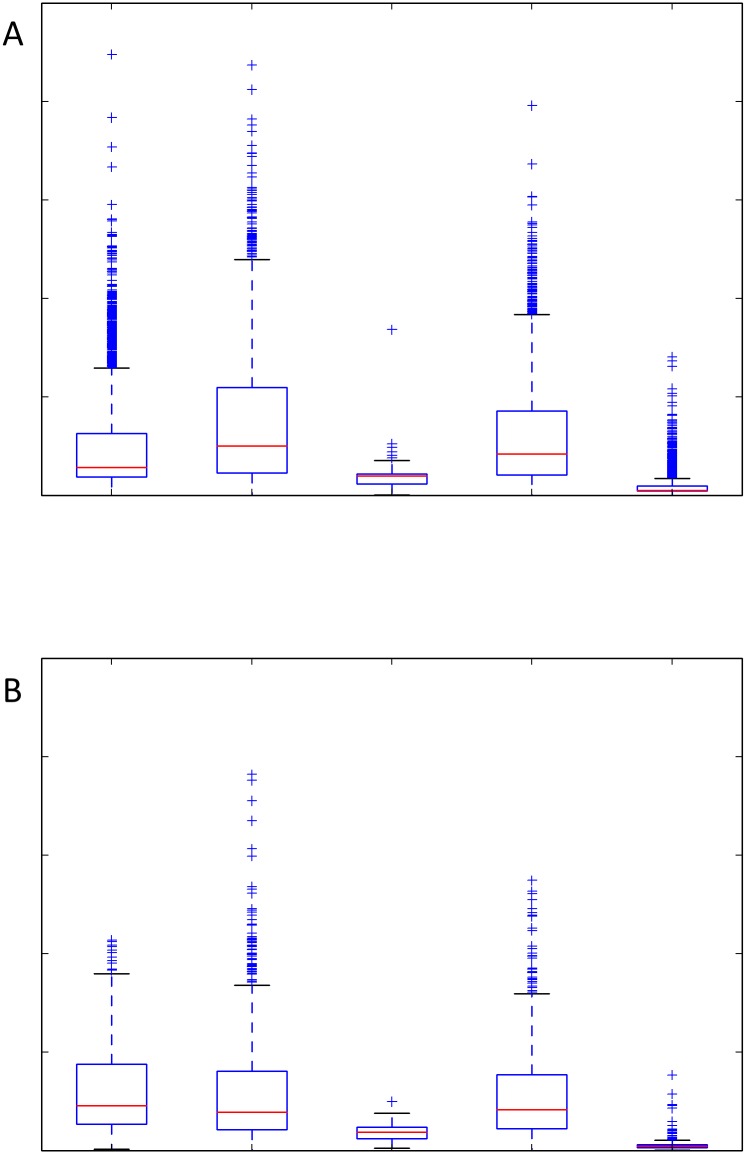
Time intervals between treatment start and testing for ADA differs between treatments. Years from treatment start to first sample tested (A), with patient numbers and median time given in S2 Table. Years from treatment start to first positive test (B), with summary information presented in S3 Table. Results were obtained from NAb assays for IFNβ and ADA assay for natalizumab.

Most patients had their first ADA test within the first two to four years of treatment (Fig 3). The longest period was noted for patients treated with IFNβ-1b-Betaferon that was introduced on the market several years before ADA testing started. The shortest time between treatment start and first test was observed for IFNβ-1b-Extavia and natalizumab for which ADA tests were available immediately.

The time to first sample (Fig 3) shows similar characteristics as time to first positive sample (Fig 3), with the group treated with IFNβ-1a i.m having the longest maximum time to first positive sample and nearly equal median (2.27 years) as IFNβ-1a s.c. (2.09 years) and IFNβ-1b-Betaferon (1.96 years) (Fig 3). The time to first positive sample for IFNβ-1b-Extavia and natalizumab was much shorter (0.94 and 0.23 years, respectively), although the low number of patients in the IFNβ-1b-Extavia group (42 patients) makes these results more uncertain than for the natalizumab-treated group (245 patients). The vast majority of the positive samples were the first sample tested (Fig 3). Patients treated with i.m. IFNβ-1a, s.c. IFNβ-1a and IFNβ-1b-Betaferon had the longest median times to the first positive sample, and thus they were administered the drug for a long time despite being ADA positive.

### Rates of ADA differ depending on IFNβ preparation

The immunogenicity of the different drug preparations varies and the same trend was confirmed in the different countries and cohorts in ranking, but not in absolute percentage. IFNβ-1a i.m. and natalizumab were the least immunogenic drugs, whereas the highest frequencies of ADA were observed for the identical IFNβ-1b preparations (Betaferon^®^ and Extavia^®^). Before the new formulation of IFNβ-1a s.c. was released in 2007, the ADA frequency was as high as for the two IFNβ-1b preparations, but the clear and lasting reduction in positive samples observed after 2007 could indicate that this formulation was less immunogenic than the previous one (Table 3).

**Table 3:** Percentage of positive samples for each treatment stratified by country. Results were obtained from NAb assays for IFNβ and ADA assay for natalizumab over the entire time of the study.

Treatment	Austria	Denmark	Germany/ Dusseldorf	Germany/ Munich	Spain	Sweden	Switzerland
**IFNβ-1a i.m.**	6.9	3.5	7.8	2.8	8.8	9.9	13.3
**IFNβ-1a s.c.**	23.4	15.0	26.9	11.5	29.4	34.0	23.6
**IFNβ-1b-Betaferon**	25.3	14.8	40.4	13.3	68.3	48.7	28.6
**IFNβ-1b-Extavia**	-	23.9	37.5	-	-	40.2	-
**Natalizumab**	6.1	5.4	-	-	-	2.5	-

### Comparison between IFNβ ADA assays

In this study, six different assays were used over time to analyze ADA against IFNβ in the different countries (Table 1). A difference in the overall proportion of ADA positive samples was observed between the assays and ranged from 10.0% for the In Vivo assay to 37.9% for the CPE assay (Table 4). The variation in ADA detection rate could reflect differences in sensitivity of the assays, but may also be influenced by the proportion of samples tested against the different IFNβ preparations as they vary in immunogenicity. In fact, the high ADA detection rate of the CPE assay could to some extent be influenced by a relatively higher proportion of samples from patients treated with the more immunogenic IFNβ-1b-Betaferon preparation compared to the less immunogenic IFNβ-1a i.m. preparation (43% vs 29%). For the iLite assay, the opposite proportions between the most immunogenic (IFNβ-1b-Betaferon and IFNβ-1b-Extavia) and least immunogenic (IFNβ-1a i.m.) preparations (28% vs 51%) may partly explain the low detection rate observed for the iLite assay (S1 Table). Nevertheless, after stratification for IFNβ preparation the same trend was observed across the assays, with IFNβ-1a i.m. being the least immunogenic and the two IFNβ-1b s.c. preparations being the most immunogenic, except for the MPA assay where the ADA frequency was highest for the IFNβ-1a s.c. preparation (Table 4).

**Table 4:** Proportion of ADA positive samples detected for each assay stratified by IFNβ preparation.

	IFNβ-1a i.m.		IFNβ-1a s.c.		IFNβ-1b-Betaferon		IFNβ-1b-Extavia		All preparations	
Assay	Positive	Total	Positive	Total	Positive	Total	Positive	Total	Positive	Total
**CPE**	3 (8.8%)	34	10 (31.3%)	32	31 (62.0%)	50	0 (n.a.)	0	44 (37.9%)	116
**LUC**	249 (9.0%)	2777	930 (35.6%)	2613	1025 (52.8%)	1943	115 (56.1%)	205	2319 (30.8%)	7538
**MPA**	221 (9.5%)	2319	1128 (33.1%)	3408	781 (29.9%)	2611	0 (n.a.)	0	2130 (25.5%)	8338
**MGA**	188 (8.5%)	2207	406 (23.9%)	1701	686 (42.8%)	1601	45 (44.1%)	102	1325 (23.6%)	5611
**iLite**	15 (5.0%)	299	26 (21.1%)	123	25 (28.4%)	88	27 (36.0%)	75	93 (15.9%)	585
**InVivo**	15 (2.8%)	540	92 (11.5%)	799	106 (13.4%)	792	0 (n.a.)	0	213 (10.0%)	2131

### Development of ADA against natalizumab after previous anti-IFNβ ADA positivity

Patients that had been tested for ADA against both IFNβ and natalizumab after treatment start were investigated to determine if ADA positivity to IFNβ was associated with subsequent development of anti-natalizumab ADA. Among patients tested for ADA against both treatments (n = 1414), 82 of 1070 (7.6%) anti-IFNβ ADA-negative patients were eventually positive for anti-natalizumab ADA. Of the 344 anti-IFNβ ADA-positive patients, 18 patients (5.2%) were also positive for ADA to natalizumab, but a statistically significant association was not present (p = 0.1467). However, due to low numbers of patients with ADA to natalizumab in both groups, an association cannot be excluded.

### Age and gender of MS patients tested positive for ADA

**Fig 4:**
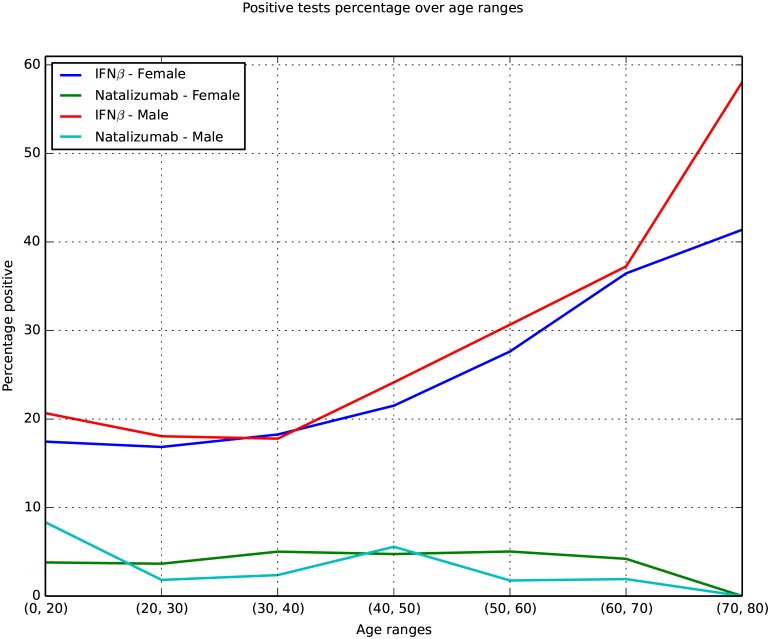
Proportion of ADA-positive tests in different age categories, stratified by treatment and gender. The number of tests given in patient age intervals for each preparation and by gender. Test numbers are given in S4 Table.

A higher rate of ADA-positive samples for the IFNβ preparations was observed with age. The proportion of ADA-positive tests was nearly twice as high in patients older than 60 years compared to those that were younger than 30 years. The percentage of ADA-positive samples was similar for men and women for both IFNβ and natalizumab (Fig 4).

## Discussion

This is the first large scale descriptive study conducted to report how ADA testing against IFNβ and natalizumab in European patients with MS has been used as routine clinical practice and as part of research studies. In this large dataset, we were able to confirm the previously observed differences in immunogenicity between the four IFNβ preparations. The decline in samples tested for ADA against the more immunogenic preparations IFNβ-1b-Betaferon and IFNβ-1a s.c. in favor of the least immunogenic preparation IFNβ-1a i.m. during the investigated time period could at least in part be due to the increased awareness of the variation in immunogenicity between the IFNβ preparations. This was previously reported for the Swedish population of NAb tested patients treated with IFNβ included here [14], but could now be confirmed in a larger cohort including other sites with established methods for routine monitoring of ADA. Nevertheless, the IFNβ preparations with the highest immunogenicity are still frequently used, indicating that ADA is far from the major factor that guides choice of treatment alternatives. For example, both IFNβ-1a s.c. and IFNβ-1b-Betaferon have been suggested to be more effective for preventing relapses in relapsing remitting MS patients than IFNβ-1a i.m. [24–26] and IFNβ-1b-Betaferon might be more effective in slowing down disease progression in secondary progressive MS patients that still have relapse activity [27]. There are also indications of that patients treated with IFNβ-1b have a greater chance of reverting to NAb negativity during continuous treatment [28,29]. Thus, even though ADA testing has become an integrated part of treatment decision for MS patients in several parts of Europe, other factors are also influencing actual clinical practice.

Assays used for ADA detection and quantification can differ in sensitivity, and the same type of assay method can vary between laboratories depending not only on differences in reagents and cell culture methods, but also on how the minimal required dilution of serum and the assay cut-point have been determined. Today there are several assays for IFNβ NAb testing and in this study five different cell-based assays and one *in vivo* assay were used. As expected, the different ADA assay methods that were used over time by the different countries and cohorts gave different rates of ADA positivity, among which the CPE assay and LUC assay detected the highest and the In Vivo assay and iLite assay detected the lowest percentage of positive samples. The variation in ADA detection indicates that the assays differ in sensitivity, which has also been previously reported for the MGA and iLite assay [21]. In addition, there are other possible factors that could account for the observed differences. Some of the variation could reflect that a greater proportion of the analyzed samples were from patients treated with the most immunogenic preparation IFNβ-1b-Betaferon (e.g. for the CPE assay) or with the least immunogenic preparation IFNβ-1a i.m. (e.g. for the iLite assay) and this has changed over time. For assays that were introduced later, the proportion of patients on less immunogenic drugs have increased, giving the impression of lower sensitivity of these assays. For the LUC assay, the relatively high percentage of ADA positive samples could be influenced by the fact that some countries using this assay first screened samples for binding ADA with an ELISA assay before testing for neutralizing ADA, resulting in an enrichment of positive samples tested with the LUC assay. The low percentage of positive samples detected by the In Vivo assay has largely to do with the definition of positivity, since a sample is only considered positive if it significantly reduces the biological effect of IFNβ. The corresponding titer in a cell-based NAb assay would be in the range of 150 TRU/mL [21,30].

Although the detection rate of ADA varied between the assays, the overall number of ADA positive samples over time as well as the already established differences in immunogenicity of the different IFNβ preparations, did not change. Nevertheless, the difference in sensitivity between ADA assay methods highlights the importance of using the same validated assay across laboratories to get comparable results when performing ADA testing. One of the first tasks of the ABIRISK project was to establish and validate assays for binding (manuscript submitted) and neutralizing ADA against IFNβ [31] and ADA against natalizumab (manuscript in progress), since methodological variations among different assays can impact the results reported between studies. The clinical relevance of low titer ADA can be questioned and therefore the most sensitive assays may not be required for routine monitoring of ADA in the clinic. In contrast, in the pharmaceutical industry it is crucial to use highly sensitive assays for immunogenicity risk assessments of newly developed drugs. In research, sensitive assays are necessary in order to detect low titers of ADA to be able to investigate the immunological processes and genetic factors that could predispose to the development of ADA.

Following previously published recommendations, it is advised to routinely test for anti-IFNβ NAbs after 12 and 24 months of treatment, and in case of positivity repeated measurements should be performed within three to six months [10,32]. However, the clinical practice of testing for NAb against IFNβ has varied between countries, ranging from being mandatory to only being recommended when NAb are suspected or provided as part of a research project. With this extensive dataset, we were able to investigate how well these recommendations were followed for the different preparations. The results showed that when there is an established routine assay for ADA detection, the recommendations for testing are followed fairly well, as seen for natalizumab for which the test was introduced simultaneously with the release of the drug on the market. However, for IFNβ there were surprisingly high numbers of patients that had been treated for many years before having an ADA test for the first time. The delayed testing could be in part due to that these patients have shown a favorable disease course, not indicating any need for treatment change. However, the observation that most patients were positive already in their first tested sample indicates that patients may have been treated for many years despite being ADA positive and most likely having had reduced effect of the treatment. Thus, even though the clinical picture might not indicate any treatment failure, the inability of the IFNβ to reach the IFN-receptor due to blocking by ADA [30,33] makes the continuous injection of IFNβ unnecessary. Considering that anti-IFNβ NAbs generally develop between 9–18 months after treatment start [34] and that the median of patients treated with the IFNβ preparations had their first ADA test after around two years of treatment, a major fraction of these patients with NAb titers high enough to reduce the biological effect of the drug could have been switched to a more effective treatment earlier. Similar to natalizumab, in the group treated with IFNβ-1b-Extavia, a preparation introduced on the market when ADA testing against IFNβ had already been implemented, the patients had their first sample tested for ADA after a median of 0.99 years which is in agreement with published recommendations [32]. Thus, ADA testing could potentially decrease unnecessary use of inefficient drugs to the benefit of both the patient and the economy of the health care system.

For natalizumab, the recommended time to first testing varied between countries from every month to six months or in case of indication of ADA. Most ADA-positive patients treated with natalizumab were already positive in their first sample taken at a median of three months after treatment start, as has been found previously [13,35] and is especially relevant for drugs that are monoclonal antibodies [36–38]. Thus, if ADA testing is in place when a drug is released, it is possible to detect positivity quite soon after treatment initiation and considerably reduce time on inefficient treatment. Apart from the unnecessary pain for the patients due to repeated injections with a drug that has reduced or no effect, the overall economical consequence for the health care system of not including ADA testing in treatment decision can be estimated from data presented in this study. Considering overall health costs there is a balance between promoting a general screening for ADA and several factors such as drug costs, costs for ADA testing, the drug efficacy and the frequency of ADA. For example, it has been suggested that routine monitoring of NAb in patients treated with IFNβ-1a i.m. may not be justifiable from an economic perspective since the frequency of NAb repeatedly have been found to be low and the number of identified ADA-positive patients switching treatment has to be compared to the cost of general ADA screening [39]. However, as an example from the data presented here, of the five individuals who had their first positive test for IFNβ-1b-Betaferon at 15 years or longer after treatment initiation, only one had previous tests, which were negative or borderline before the present positive test. The other four patients were positive in their first tests, and therefore may have been positive many years earlier. One reason for this long time before testing could be that the patients were only tested if there was any indication of treatment failure. This might be costly though. With an average of 17.65 years until the first positive test in these patients, and assuming they became positive within the first two years of treatment [34], they have had less efficient treatment for over a decade. With the yearly cost of IFNβ-1b-Betaferon treatment ranging from $16,000–$25,000 [40,41], the total cost of a patient found positive after two years of treatment would be up to $391,250 (15.65 x $25,000) per person. These types of overall health care costs and benefits have to be considered for all current used BPs individually in conjunction with costs for ADA screening, but might be especially beneficial for very expensive drugs if these are monoclonal antibodies where ADA can be detected early after treatment initiation.

Extrapolating the reasoning to a European scale by using data on prices of therapies in Sweden from 2016, combined with the data on positive tests from Fig 3, gives an estimate of approximate cost savings created by testing patients for anti-IFNβ NAbs as well as the possible costs incurred by less stringent testing regiments previously. By using the current prices of each therapy and the extrapolated cost for the total period and per 1000 MS patients with a similar distribution of treatments over the 18 years of data in Fig 3, €35,106,852 (1€ = 0.10745 SEK) was spent on therapies after NAb positivity was expected. If half of these NAb-positive patients given therapy after one year had ineffective treatment due to high titers of neutralizing ADA, a total of €17,553,426 was spent that could have been redirected toward more effective health care or other therapeutics. This corresponds to €418,896 per 1000 MS patients each year that was misdirected, a figure that can be used to estimate potential national savings in health care costs by introducing ADA routine testing in relation to number of MS patients on treatment, and compared to cost of screening for ADA. In Sweden, data from the national MS registry shows that over 3,500 MS patients have been treated with IFNβ each year the last 10 years, and thus accumulated cost of €14 million could have been saved by having a highly efficient NAb testing routine and strict compliance to the recommendations.

Another means of avoiding patients becoming inefficiently treated due to ADA is to identify risk factors predicting susceptibility to ADA development and consider these already before start of therapy. In this study we assessed whether MS patients that have been previously positive for anti-IFNβ NAb were more likely to develop anti-natalizumab ADA. As previously reported in a Danish study on 318 consecutive patients treated with both IFNβ and natalizumab [42], we did not find an association between development of anti-IFNβ NAb and ADA against natalizumab.

We observed a higher rate of ADA-positive tested samples for the IFNβ preparations with older age. Even though this study was not appropriately designed to detect age and gender as true risk factors for ADA, it is clinically useful to see that samples from older patients might give a higher ADA frequency compared to those reported from clinical trials. To specifically address the impact of age and gender, as well as other factors on the risk of developing ADA against IFNβ and natalizumab, the focus of the study should be on a cohort of naive patients with tests performed during the window of time for ADA appearance.

In conclusion, by characterizing ADA test results from the last decade from several countries in Europe, we have shown that by having a strategy for ADA testing in place when a drug is released it is possible to ascertain positivity earlier which could promote therapy changes and shorten the time that ADA-positive patients stay on less effective treatments. Thus, since the immunogenicity of BPs can be detected by ADA tests, there is room for improving the efficacy in health care by integrating this aspect in clinical practice.

## Supporting information

S1 Fig Flow chart of quality control of the data. (TIF)

S1 Table Proportion of samples per IFNβ preparation tested by each assay. (DOCX)

S2 Table Numbers of patients and median years to first test by preparation. (DOCX)

S3 Table Patient numbers and test information by preparation. (DOCX)

S4 Table Number of tests by patient age ranges. (DOCX)

S1 File List of ABIRISK Consortium partners and members. (DOCX)

## References

[1] The PRISMS (Prevention of Relapses and Disability by Interferon-beta-1a Subcutaneously in Multiple Sclerosis) Study Group. PRISMS-4: Long-term efficacy of interferon-beta-1a in relapsing MS. Neurology. 2001;56: 1628–1636. 11425926 10.1212/wnl.56.12.1628

[2] The IFNB Multiple Sclerosis Study Group. Interferon beta-1b is effective in relapsing-remitting multiple sclerosis. I. Clinical results of a multicenter, randomized, double-blind, placebo-controlled trial. Neurology. 1993;43: 655–661. 8469318 10.1212/wnl.43.4.655

[3] Jacobs LD, Cookfair DL, Rudick RA, Herndon RM, Richert JR, Salazar AM, et al Intramuscular interferon beta-1a for disease progression in relapsing multiple sclerosis. The Multiple Sclerosis Collaborative Research Group (MSCRG). Ann Neurol. 1996;39: 285–294. 10.1002/ana.410390304 8602746

[4] Deisenhammer F. Neutralizing antibodies to interferon-beta and other immunological treatments for multiple sclerosis: prevalence and impact on outcomes. CNS Drugs. 2009;23: 379–96. 10.2165/00023210-200923050-00003 19453200

[5] Link J, Ryner Lundkvist M, Fink K, Hermanrud C, Lima I, Brynedal B, et al Human leukocyte antigen genes and interferon beta preparations influence risk of developing neutralizing anti-drug antibodies in multiple sclerosis. PLoS One. 2014;9. 10.1371/journal.pone.0090479 PMC3946519 24608124

[6] Calabresi PA, Giovannoni G, Confavreux C, Galetta SL, Havrdova E, Hutchinson M, et al The incidence and significance of anti-natalizumab antibodies: Results from AFFIRM and SENTINEL. Neurology. 2007;69: 1391–1403. 10.1212/01.wnl.0000277457.17420.b5 17761550

[7] Sørensen PS, Hyldgaard Jensen PE, Haghikia A, Lundkvist M, Vedeler C, Sellebjerg F, et al Occurrence of antibodies against natalizumab in relapsing multiple sclerosis patients treated with natalizumab. Mult Scler. 2011;17: 1074–1078. 10.1177/1352458511404271 21511692

[8] Szalma S, Koka V, Khasanova T, Perakslis ED. Effective knowledge management in translational medicine. J Transl Med. 2010;8: 68 10.1186/1479-5876-8-68 20642836 PMC2914663

[9] R Development Core Team R, R Core Team. R: A language and environment for statistical computing. R A Lang Environ Stat Comput. 2014;0: 409.

[10] Polman CH, Bertolotto A, Deisenhammer F, Giovannoni G, Hartung H-P, Hemmer B, et al Recommendations for clinical use of data on neutralising antibodies to interferon-beta therapy in multiple sclerosis. Lancet Neurol. 2010;9: 740–50. 10.1016/S1474-4422(10)70103-4 20610349

[11] Pachner AR. An improved ELISA for screening for neutralizing anti-IFN-beta antibodies in MS patients. Neurology. 2003;61: 1444–6. 14638976 10.1212/01.wnl.0000094198.37489.11

[12] Sominanda A, Rot U, Suoniemi M, Deisenhammer F, Hillert J, Fogdell-Hahn A. Interferon beta preparations for the treatment of multiple sclerosis patients differ in neutralizing antibody seroprevalence and immunogenicity. Mult Scler. 2007;13: 208–14. 10.1177/1352458506070762 17439886

[13] Jensen PEH, Koch-Henriksen N, Sellebjerg F, Sørensen PS. Prediction of antibody persistency from antibody titres to natalizumab. Mult Scler. 2012;18: 1493–9. 10.1177/1352458512441688 22454098

[14] Jungedal R, Lundkvist M, Engdahl E, Ramanujam R, Westerlind H, Sominanda A, et al Prevalence of anti-drug antibodies against interferon beta has decreased since routine analysis of neutralizing antibodies became clinical practice. Mult Scler. 2012;18: 1775–81. 10.1177/1352458512446036 22551640

[15] Massart C, Gibassier J, Oger J, Le Page E, Edan G. Neutralizing antibodies to interferon beta in multiple sclerosis: Analytical evaluation for validation of a cytopathic effect assay. Clin Chim Acta. 2007;377: 185–191. 10.1016/j.cca.2006.09.021 17123498

[16] Lam R, Farrell R, Aziz T, Gibbs E, Giovannoni G, Grossberg S, et al Validating parameters of a luciferase reporter gene assay to measure neutralizing antibodies to IFNbeta in multiple sclerosis patients. J Immunol Methods. 2008;336: 113–8. 10.1016/j.jim.2008.03.014 18511063

[17] Hegen H, Millonig A, Bertolotto A, Comabella M, Giovanonni G, Guger M, et al Early detection of neutralizing antibodies to interferon-beta in multiple sclerosis patients: binding antibodies predict neutralizing antibody development. Mult Scler. 2014;20: 577–87. 10.1177/1352458513503597 24009164

[18] Farrell R, Kapoor R, Leary S, Rudge P, Thompson A, Miller D, et al Neutralizing anti-interferon beta antibodies are associated with reduced side effects and delayed impact on efficacy of Interferon-beta. Mult Scler. 2008;14: 212–218. 10.1177/1352458507082066 17986510

[19] Hoffmann S, Cepok S, Grummel V, Lehmann-Horn K, Hackermüller J, Hackermueller J, et al HLA-DRB1*0401 and HLA-DRB1*0408 are strongly associated with the development of antibodies against interferon-beta therapy in multiple sclerosis. Am J Hum Genet. 2008;83: 219–27. 10.1016/j.ajhg.2008.07.006 18656179 PMC2495071

[20] Lallemand C, Meritet JF, Erickson R, Grossberg SE, Roullet E, Lyon-Caen O, et al Quantification of neutralizing antibodies to human type I interferons using division-arrested frozen cells carrying an interferon-regulated reporter-gene. J Interf Cytokine Res. 2008;28: 393–404. 10.1089/jir.2007.0142 18593334

[21] Hermanrud C, Ryner ML, Engdahl E, Fogdell-Hahn A. Anti-interferon beta antibody titers strongly correlate between two bioassays and in vivo biomarker expression, and indicates that a titer of 150 TRU/mL is a biologically functional cut-point. J Interferon Cytokine Res. 2014;34: 498–504. 10.1089/jir.2013.0097 24444338

[22] Kawade Y. Quantitation of neutralization of interferon by antibody. Methods Enzymol. 1986;119: 558–73. 2429157 10.1016/0076-6879(86)19076-8

[23] R Core Team. R: A Language and Environment for Statistical Computing. R Found Stat Comput Vienna, Austria 2015; http://www.r-project.org/

[24] Durelli L, Verdun E, Barbero P, Bergui M, Versino E, Ghezzi A, et al Every-other-day interferon beta-1b versus once-weekly interferon beta-1a for multiple sclerosis: results of a 2-year prospective randomised multicentre study (INCOMIN). Lancet. 2002;359: 1453–1460. 11988242 10.1016/s0140-6736(02)08430-1

[25] Panitch H, Goodin D, Francis G, Chang P, Coyle P, O’Connor P, et al Benefits of high-dose, high-frequency interferon beta-1a in relapsing-remitting multiple sclerosis are sustained to 16 months: Final comparative results of the EVIDENCE trial. J Neurol Sci. 2005;239: 67–74. 10.1016/j.jns.2005.08.003 16169561

[26] Koch-Henriksen N, Sørensen P, Christensen T, Frederiksen J, Ravnborg M, Jensen K, et al A randomized study of two interferon-beta treatments in relapsing-remitting multiple sclerosis. Neurology. 2006;66: 1056–1060. 10.1212/01.wnl.0000204018.52311.ec 16510769

[27] Kappos L. Effect of drugs in secondary disease progression in patients with multiple sclerosis. Mult Scler. 2004;10 Suppl 1: S46-54–5. Available: http://www.ncbi.nlm.nih.gov/pubmed/15218809 10.1191/1352458504ms1030oa 15218809

[28] Gneiss C, Reindl M, Lutterotti A, Ehling R, Egg R, Khalil M, et al Interferon-beta: the neutralizing antibody (NAb) titre predicts reversion to NAb negativity. Mult Scler. 2004;10: 507–510. 15471365 10.1191/1352458504ms1074oa

[29] Sorensen PS, Koch-Henriksen N, Ross C, Clemmesen KM, Bendtzen K. Appearance and disappearance of neutralizing antibodies during interferon-beta therapy. Neurology. 2005;65: 33–39. 10.1212/01.WNL.0000166049.51502.6A 15888603

[30] Sominanda A, Hillert J, Fogdell-Hahn A. In vivo bioactivity of interferon-beta in multiple sclerosis patients with neutralising antibodies is titre-dependent. J Neurol Neurosurg Psychiatry. 2008; 10.1136/jnnp.2007.122549 17911184

[31] Hermanrud C, Ryner M, Luft T, Jensen PE, Ingenhoven K, Rat D, et al Development and validation of cell-based luciferase reporter gene assays for measuring neutralizing anti-drug antibodies against interferon beta. J Immunol Methods. 2016;430: 1–9. 10.1016/j.jim.2016.01.004 26779831

[32] Sørensen PS, Deisenhammer F, Duda P, Hohlfeld R, Myhr KM, Palace J, et al Guidelines on use of anti-IFN-β antibody measurements in multiple sclerosis: Report of an EFNS Task Force on IFN-β antibodies in multiple sclerosis. European Journal of Neurology. 2005 pp. 817–827. 10.1111/j.1468-1331.2005.01386.x 16241970

[33] Hesse D, Sellebjerg F, Sorensen PS. Absence of MxA induction by interferon beta in patients with MS reflects complete loss of bioactivity. Neurology. 2009;73: 372–377. 10.1212/WNL.0b013e3181b04c98 19652141

[34] Hesse D, Sørensen PS. Using measurements of neutralizing antibodies: the challenge of IFN-beta therapy. Eur J Neurol. 2007;14: 850–9. 10.1111/j.1468-1331.2007.01769.x 17662004

[35] Lundkvist M, Engdahl E, Holmén C, Movérare R, Olsson T, Hillert J, et al Characterization of anti-natalizumab antibodies in multiple sclerosis patients. Mult Scler. 2013;19: 757–64. 10.1177/1352458512462920 23045379

[36] Hoxha A, Calligaro A, Tonello M, Ramonda R, Carletto A, Paolazzi G, et al The clinical relevance of early anti-adalimumab antibodies detection in rheumatoid arthritis, ankylosing spondylitis and psoriatic arthritis: A prospective multicentre study. Jt Bone Spine. 2016;83: 167–171. 10.1016/j.jbspin.2015.04.020 26750762

[37] Geborek BK, Svenson P, Larsson M, Mc K, Saxne T. Individualized monitoring of drug bioavailability and immunogenicity in rheumatoid arthritis patients treated with the tumor necrosis factor alpha inhibitor infliximab. Arthritis Rheum. 2006;54: 3782–9. 10.1002/art.22214 17133559

[38] Bartelds GM, Krieckaert CLM, Nurmohamed MT, van Schouwenburg PA, Lems WF, Twisk JWR, et al Development of antidrug antibodies against adalimumab and association with disease activity and treatment failure during long-term follow-up. JAMA. 2011;305: 1460–8. 10.1001/jama.2011.406 21486979

[39] Walter E, Deisenhammer F. Socio-economic aspects of the testing for antibodies in MS-patients under interferon therapy in Austria: a cost of illness study. Mult Scler Relat Disord. 2014;3: 670–7. 10.1016/j.msard.2014.09.003 25891545

[40] Castrop F, Haslinger B, Hemmer B, Buck D. Review of the pharmacoeconomics of early treatment of multiple sclerosis using interferon beta. Neuropsychiatr Dis Treat. 2013;9: 1339–1349. 10.2147/NDT.S33949 24072971 PMC3783501

[41] Newton AN, Stica CM. A Comprehensive Cost-Effectiveness Analysis of Treatments for Multiple Sclerosis. Int J MS Care. 2011;13: 128–135. 10.7224/1537-2073-13.3.128 24453716 PMC3882969

[42] Sørensen PS, Koch-Henriksen N JP. Neutralizing antibodies against interferon-beta do not predispose antibodies against natalizumab. Neurology. 2011;76: 759–760. 10.1212/WNL.0b013e31820d62a4 21339504

