# Supplementary figures and images for: Clinical practice of analysis of anti-drug antibodies against interferon beta and natalizumab in multiple sclerosis patients in Europe: A descriptive study of test results
Source: PLoS One. 2017 Feb 7;12(2):e0170395. doi: 10.1371/journal.pone.0170395 (PMC5295710; doi:10.1371/journal.pone.0170395)

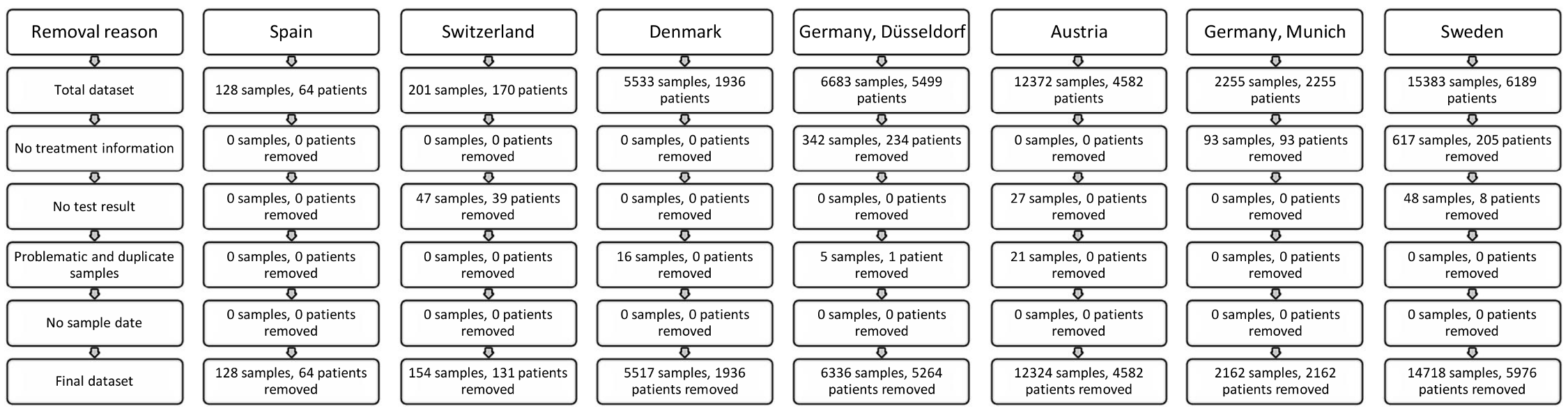

Supplement: S1 Fig — (TIF) [file pone.0170395.s001.tif]
