# Supplementary material for: Clinical practice of analysis of anti-drug antibodies against interferon beta and natalizumab in multiple sclerosis patients in Europe: A descriptive study of test results
Source: PLoS One. 2017 Feb 7;12(2):e0170395. doi: 10.1371/journal.pone.0170395 (PMC5295710; doi:10.1371/journal.pone.0170395)
Supplement: S1 Table — (DOCX) [file pone.0170395.s002.docx]

**S1 Table. Proportion of samples per IFNβ preparation tested by each assay.**

|  | **IFNβ-1a i.m.** | | **IFNβ-1a s.c.** | | **IFNβ-1b-Betaferon** | | **IFNβ-1b-Extavia** | |
| --- | --- | --- | --- | --- | --- | --- | --- | --- |
| **Assay** | **Positive** | **Total** | **Positive** | **Total** | **Positive** | **Total** | **Positive** | **Total** |
| **CPE** | 3 (7%) | 34 (29%) | 10 (23%) | 32(28%) | 31(70%) | 50 (43%) | 0 (n.a.) | 0 (n.a.) |
| **LUC** | 249 (11%) | 2777 (37%) | 930 (40%) | 2613 (35%) | 1025 (44%) | 1943 (26%) | 115 (5%) | 205 (3%) |
| **MPA** | 221 (10%) | 2319 (28%) | 1128 (53%) | 3408 (41%) | 781 (37%) | 2611 (31%) | 0 (n.a.) | 0 (n.a.) |
| **MGA** | 188 (14%) | 2207 (39%) | 406 (31%) | 1701(30%) | 686 (52%) | 1601 (29%) | 45 (3%) | 102 (2%) |
| **iLite** | 15 (16%) | 299 (51%) | 26 (28%) | 123 (21%) | 25 (27%) | 88 (15%) | 27 (29%) | 75 (13%) |
| **InVivo** | 15 (7%) | 540 (25%) | 92 (43%) | 799 (37%) | 106 (50%) | 792 (37%) | 0 (n.a.) | 0 (n.a.) |
