# Supplementary material for: Clinical practice of analysis of anti-drug antibodies against interferon beta and natalizumab in multiple sclerosis patients in Europe: A descriptive study of test results
Source: PLoS One. 2017 Feb 7;12(2):e0170395. doi: 10.1371/journal.pone.0170395 (PMC5295710; doi:10.1371/journal.pone.0170395)
Supplement: S2 Table — (DOCX) [file pone.0170395.s003.docx]

**S2 Table. Numbers of patients and median years to first test by preparation.**

|  | **IFNβ-1a i.m.** | **IFNβ-1b-Betaferon** | **IFNβ-1b-Extavia** | **IFNβ-1a s.c.** | **Natalizumab** |
| --- | --- | --- | --- | --- | --- |
| Number of patients | 3873 | 2908 | 110 | 3765 | 1760 |
| Median years to first test | 1.41 | 2.51 | 0.99 | 2.11 | 0.25 |

*^*^ Note that a patient may have been tested for more than one treatment*
