# Supplementary material for: Clinical practice of analysis of anti-drug antibodies against interferon beta and natalizumab in multiple sclerosis patients in Europe: A descriptive study of test results
Source: PLoS One. 2017 Feb 7;12(2):e0170395. doi: 10.1371/journal.pone.0170395 (PMC5295710; doi:10.1371/journal.pone.0170395)
Supplement: S3 Table — (DOCX) [file pone.0170395.s004.docx]

**S3 Table. Patient numbers and test information by preparation.**

|  | **IFNβ-1a i.m.** | **IFNβ-1b-Betaferon** | **IFNβ-1b-Extavia** | **IFNβ-1a s.c.** | **Natalizumab** |
| --- | --- | --- | --- | --- | --- |
| Number of patients | 229 | 924 | 42 | 888 | 245 |
| Median years to first positive test | 2.27 | 1.96 | 0.94 | 2.09 | 0.23 |
| No previous test on the same preparation (no. of patients, % of all tested) | 168 (73.4) | 839 (90.8) | 37 (88.1) | 802 (90.3) | 178 (72.6) |
| Previous negative tests on the same preparation (no. of patients, % of all tested) | 59 (25.8) | 79 (8.5) | 1 (2.4) | 81 (9.1) | 10 (4.1) |
| Previous negative tests on other treatment (no. of patients, % of all tested) | 2 (0.9) | 6 (0.6) | 4 (9.5) | 5 (0.6) | 57 (23.3) |
