# Supplementary material for: Clinical practice of analysis of anti-drug antibodies against interferon beta and natalizumab in multiple sclerosis patients in Europe: A descriptive study of test results
Source: PLoS One. 2017 Feb 7;12(2):e0170395. doi: 10.1371/journal.pone.0170395 (PMC5295710; doi:10.1371/journal.pone.0170395)
Supplement: S4 Table — (DOCX) [file pone.0170395.s005.docx]

**S4 Table. Number of tests by patient age ranges.**

| Age range (year) | 0-20 | 20-30 | 30-40 | 40-50 | 50-60 | 60-70 | 70-80 |
| --- | --- | --- | --- | --- | --- | --- | --- |
| Number of tests | 712 | 7030 | 12658 | 12434 | 5666 | 1321 | 96 |
